# Rethinking Warm-Up in Overhead Exercise: Acute Shoulder Responses to a Strength- and Mobility-Oriented Protocol in Youth Athletes

**DOI:** 10.3390/sports14050203

**Published:** 2026-05-15

**Authors:** Andrea Pagliaro, Alessia Boatta, Anna Alioto, Roberta Cottone, Domenico Nuzzo, Pasquale Picone, Cristina Cortis, Andrea Fusco, Magdalena Dzitkowska-Zabielska, Giuseppe Messina, Patrizia Proia

**Affiliations:** 1 Sport and Exercise Sciences Research Unit, Department of Psychology, Educational Science and Human, University of Palermo, 90128 Palermo, Italy; andrea.pagliaro@unipa.it (A.P.); anna.alioto@unipa.it (A.A.); roberta.cottone@univr.it (R.C.); patrizia.proia@unipa.it (P.P.); 2 Department of Human Sciences and Promotion of the Quality of Life, San Raffaele University, 00166 Rome, Italy; alessia.boatta@uniroma5.it; 3 Institute for Biomedical Research and Innovation, National Research Council (CNR), 90146 Palermo, Italy; domenico.nuzzo@cnr.it (D.N.); pasquale.picone@cnr.it (P.P.); 4 Department of Neurosciences, Biomedicine and Movement Sciences, University of Verona, 37129 Verona, Italy; 5 Department of Human Sciences, Society and Health, University of Cassino and Lazio Meridionale, 03043 Cassino, Italy; c.cortis@unicas.it; 6 Department of Medicine and Aging Sciences, University “G. d’Annunzio” of Chieti-Pescara, 66100 Chieti, Italy; andrea.fusco@unich.it; 7 Faculty of Physical Education, Gdansk University of Physical Education and Sport, 80-336 Gdansk, Poland; magdalena.dzitkowska-zabielska@awf.gda.pl

**Keywords:** overhead athletes, youth athletes, shoulder warm-up, upper extremity performance, handgrip strength, CKCUES

## Abstract

Overhead sports place high demands on the shoulder complex, making warm-up specificity relevant for acute readiness. This randomized controlled pilot trial compared the immediate effects of a shoulder-specific warm-up with a habitual routine in 24 youth competitive overhead athletes (14–20 years), allocated to an experimental group (EG = 12) and a habitual warm-up group (SWG = 12). The warm-up protocol was administered bilaterally to both shoulders, whereas outcome measurements were collected unilaterally, with each shoulder tested separately. Assessments were performed before and immediately after the warm-up protocol. Outcome measures included shoulder flexion range of motion (ROM), handgrip strength, Closed Kinetic Chain Upper Extremity Stability (CKCUES) performance, and post-warm-up Rating of Perceived Exertion (RPE; Borg CR-10). A significant group-by-time interaction was found for right shoulder flexion ROM (*p* = 0.003, η^2^p = 0.346), with a significant increase in the EG from baseline to post-test (*p* = 0.008). No significant effects were observed for left shoulder flexion ROM, handgrip strength, or CKCUES performance. Post-warm-up RPE was statistically significant in the EG compared to the SWG (*p* = 0.041). These preliminary findings may suggest the potential practical value of more targeted warm-up strategies in overhead sports, while larger longitudinal studies are needed to confirm their broader functional relevance.

## 1. Introduction

### 1.1. Shoulder Function in Overhead Sports

In sports characterized by repeated overhead actions (such as water polo, weightlifting, rhythmic gymnastics, and shot put), shoulder efficiency does not depend solely on joint range of motion (ROM), but rather emerges from the coordinated interaction between strength, neuromuscular control, and movement perception [1]. The athlete’s ability to stabilize the scapulohumeral joint at ranges of maximal mechanical load represents a fundamental factor for both performance and injury prevention [2].

Although many preparation and prevention protocols include shoulder mobility and strengthening exercises, these are often relegated to the final phases of the training session, potentially limiting their immediate transfer to sport-specific performance [3,4,5].

A more integrated approach may consider shoulder function as part of a dynamic and interconnected system, in which local responses are influenced by the interaction between mobility, strength, and motor organization.

Within this perspective, biotensegrity may provide a theoretical framework to conceptualize the musculoskeletal system as an integrated network, while avoiding direct mechanistic conclusions based on this model [6,7]. The increasing specialization of young athletes further highlights the need for specific, sustainable strategies that support performance and may contribute to reducing injury risk over time [8].

### 1.2. Warm-Up Rationale and Outcome Selection

In this context, the warm-up represents a low-cost intervention, performed daily and capable of exerting a meaningful impact on joint and neuromuscular function [9]. However, the literature to date reports heterogeneous findings regarding its effectiveness, likely due to differences in exercise and innovative tool selection, specificity, and intended training objectives [10]. For this reason, targeted warm-up protocols consistent with the biomechanical demands of overhead sports may be more appropriate than generic whole-body activation routines [11]. The rationale of the present approach is that preparing the shoulder complex through controlled mobility, end-range activation, and stabilization-oriented exercises may acutely influence shoulder excursion and upper-extremity functional performance [12,13,14].

In line with this rationale, shoulder flexion ROM was selected as the main mobility outcome because it was the variable most directly aligned with the biomechanical focus of the proposed warm-up protocol, which emphasized arm elevation, overhead positioning, and scapulohumeral control during Y-pattern and end-range movements. Although internal rotation, external rotation, and scapular-specific assessments are also highly relevant in overhead athletes, shoulder flexion was prioritized in the present pilot study because it provided a feasible and standardized mobility measure that closely reflected the immediate movement demands of the protocol. The CKCUES was included as a field-based functional test related to upper-limb stability, whereas handgrip strength was included as an exploratory and practical indicator of upper-limb force expression, rather than as a direct mechanistic target of the warm-up. From a perceptual perspective, Borg CR-10 was included to capture the athletes’ immediate perceived response to the warm-up, rather than as a direct measure of proprioception, scapular control, or movement perception [15,16].

### 1.3. Aim of Study

Therefore, this pilot study aimed to compare the acute effects of an active warm-up based on mobility and strength exercises specific to the scapulohumeral joint with those of a traditional warm-up in young overhead competitive athletes. Specifically, this study evaluated changes in shoulder mobility, upper-extremity functional performance, and perceived response, with the broader objective of contributing to the development of more targeted, sustainable, and performance-oriented warm-up strategies.

## 2. Materials and Methods

### 2.1. Study Design

This study was conducted as a randomized controlled pilot trial. Participants were randomly assigned to a standard warm-up group (SWG), which performed their habitual warm-up routine, and an experimental group (EG), which completed the shoulder-specific warm-up protocol developed by the investigators. Outcome measures were collected at two time points: at baseline, before the warm-up (T0), and immediately after warm-up (T1). This pre–post approach was used to assess the immediate responses to the warm-up and to identify short-term changes in joint mobility, and perceptual responses [17]. All assessments were conducted in the training environment where the athletes normally practice in order to preserve ecological validity and minimize potential environmental influences. Testing was completed within a single session on the same day for each participant. The study was designed to examine, within an ecologically valid and sport-specific context, whether a shoulder-focused warm-up, compared with a habitual routine, could acutely improve shoulder readiness for performance [18]. This rationale was based on the premise that targeted mobility and functional preparation may better prepare the shoulder for overhead tasks [19].

### 2.2. Participants

A total of 30 competitive athletes were screened for eligibility. Of these, 24 athletes met the eligibility criteria and voluntarily participated in this pilot study (8 males, 16 females). Participants were randomly allocated to the SWG (*n* = 12), performing their habitual warm-up routine and the EG (*n* = 12), performing the strength- and mobility-based warm-up protocol proposed by the investigators.

Participants were randomly allocated using the Wheel of Names web-based random picker (wheelofnames.com accessed on 7 July 2025). Each eligible participant was assigned an anonymized identification code, and all codes were entered into the software. The wheel was spun sequentially to select participants for the experimental group; after each spin, the selected code was removed from the list to avoid duplicate selection. This procedure was repeated until 12 participants had been allocated to the experimental group. The remaining 12 participants were assigned to the standard warm-up group by exclusion. Group allocation was not disclosed to the evaluators during the assessments. Random allocation was used to reduce the risk that sport-modality-related characteristics would be systematically concentrated in one group.

All athletes were actively competing at a competitive level and were training regularly at the time of data collection. Participants were recruited from different overhead sport modalities. The rationale for including athletes from different disciplines was based on the shared biomechanical demands placed on the shoulder complex, particularly repeated shoulder elevation, scapular control, and upper-limb stabilization, rather than on a single sport-specific technical gesture. Limb dominance was self-reported by each participant. Most participants reported right-side dominance, with 22 athletes being right-side dominant (91.7%) and 2 athletes being left-side dominant (8.3%).

Inclusion criteria were (a) competitive athletic status; (b) age between 14 and 20 years; and (c) the absence of current or recent upper-limb injuries, with specific reference to the shoulder complex. All participants were injury-free at the time of assessment, particularly with respect to the scapulohumeral joint. Recruitment was limited by athletes’ competition commitments, which reduced their availability for participation.

Written informed consent was obtained from the parents or legal guardians of all participants under 18 years of age prior to study participation. The study was conducted according to the Declaration of Helsinki and was approved by the ethics committee of the University of Palermo 1, (Approval No. 325/2025), Department of Psychological, Educational and Movement Sciences, University of Palermo.

Anthropometric characteristics of the participants (age, height, and body mass) are reported in Table 1.

### 2.3. Assessment

All assessments were carried out in the athletes’ usual training facilities at Palermo Sport & Performance and A.S.D. Azzurra Palermo, Italy. Conducting the evaluations in a familiar environment was intended to limit context-related influences on sensorimotor control and to obtain measurements that more accurately reflect the athletes’ true performance capacity. Upon arrival, participants performed assessments at T0 using a fixed and standardized sequence: the Gyko inertial system (Microgate, Bolzano, Italy) was initially used to assess scapulohumeral joint range of motion. This was followed by the Handgrip strength test (KERN MAP Version 1.2 08/2012, KERN & Sohn GmbH, Balingen, Germany Hand Grip Dynamometer) evaluating left and right hand separately and, subsequently, by the Closed Kinetic Chain Upper Extremity Stability Test (CKCUES) [20,21,22]. For both shoulder flexion ROM and handgrip strength, the right and left upper limbs were assessed separately, with one limb tested at a time.

Perceived exertion was assessed using the Borg CR-10 category-ratio scale (0–10). Participants were familiarized with the scale anchors and asked to rate their perceived effort immediately after completion of the warm-up protocol. In the present study, the Borg CR-10 score was used as an indicator of the internal load and perceived response associated with the preparatory stimulus (e.g., perceived activation, satisfaction, and sense of shoulder freedom) [23,24].

After completion of the T0 assessments, participants-previously allocated to either the EG or SWG performed their respective warm-up protocols. The EG completed the strength-based warm-up, whereas the SWG carried out their habitual warm-up routine. Immediately afterward, all participants performed the T1 assessment in the same order as at baseline. The same evaluators performed all assessments at both time points in order to ensure procedural consistency. Although they did not know the participants personally, they were formally blinded to group allocation. To reduce measurement variability, all participants were assessed using the same order, and under the same standardized procedures at T0 and T1. Handgrip strength and CKCUES were included as complementary field-based outcomes.

Handgrip strength was not intended to represent a direct measure of shoulder-specific function, but rather a rapid and standardized indicator of maximal isometric upper-limb force expression. The CKCUES test was selected because it assesses upper-limb dynamic stability, trunk contribution, and the ability to tolerate load through the upper extremity in a closed kinetic chain. Although CKCUES does not reproduce a specific overhead sport gesture, these components are relevant to field-based upper-limb functional assessment in overhead athletes. A detailed overview of the assessment tools, measured variables, and their intended purpose is provided in Table 2.

### 2.4. Warm-Up Protocols

#### 2.4.1. Strength-Based Warm-Up

The strength-based warm-up protocol was administered bilaterally to the EG following a predefined and progressive sequence aimed at preparing the shoulder complex for overhead demands. The protocol progressed from low-intensity preparatory exercises to more specific activation and stabilization tasks, while maintaining a low controlled intensity throughout. This approach was intended to promote movement quality and shoulder preparation without inducing fatigue before the post-warm-up assessments. Exercise order, volume, duration, repetitions, and rest intervals were standardized across participants. Exercises were performed in the order reported in Table 3, and each exercise had to be completed before progressing to the next.

The protocol began with foam rolling using a lacrosse ball applied to the pectoral region [25,26]. This initial phase was included to facilitate shoulder flexion mobility and to improve tissue readiness before subsequent movements and testing [27]. Following foam rolling, athletes performed shoulder Controlled Articular Rotations (CARs). CARs consist of active, controlled, end-range rotational movements executed through the largest pain-free range of motion [28,29]. This exercise was included to promote active shoulder mobility, motor control, and joint awareness, supporting optimal scapulohumeral mechanics. The warm-up then progressed to Y-hurdle raises performed in a prone position on a bench inclined at 45°. From this position, athletes raised their arms in a Y-shaped pattern with the elbows extended, focusing on slow and controlled arm elevation. This exercise was included to enhance scapulohumeral control during shoulder elevation, with particular emphasis on the coordinated activation of the lower trapezius and serratus anterior. These muscles play a key role in optimizing scapular mechanics during overhead movements [30]. As the final exercise, the Y isometric hold was performed in a supine position. This exercise shared the same biomechanical objectives as the Y-hurdle raises but was executed isometrically. Athletes maintained the Y-shaped arm position while focusing on scapular retraction, upward rotation, and posterior tilt, minimizing compensatory movements [31]. The inclusion of an isometric contraction was intended to promote controlled muscle activation and scapular stabilization at a joint position relevant to overhead activities [32,33]. All specific details are reported in Table 3.

#### 2.4.2. Traditional Warm-Up

Participants assigned to the SWG performed their habitual warm-up routine, as normally used before training. This active comparison condition was selected to reflect usual preparatory practice in the athletes’ training setting, rather than an artificial no-warm-up condition. This allowed us to examine whether the shoulder-focused protocol produced additional acute effects compared with the athletes’ habitual routine.

The traditional warm-up consisted of general activation and mobility exercises and was not specifically designed to target shoulder strength or scapular control [34]. Exercises were performed in the same order and manner routinely adopted by the athletes prior to training. A general overview of the exercises included in the traditional warm-up is reported in Table 4.

### 2.5. Statistical Analysis

Data are presented as the mean ± standard deviation (SD). The normality of data distribution was assessed using the Shapiro–Wilk test. For each outcome measure, ANOVA (2 × 2) was performed, with group as the between-subjects factor and time (T0 vs. T1) as the within-subjects factor. When significant effects were detected in the ANOVA, Bonferroni-adjusted post hoc comparisons were performed. Effect sizes were reported as partial eta squared (η^2^_p_). Statistical significance was set at *p* < 0.05. Borg CR-10 scores were only collected at T1 and were therefore analyzed separately. Given the ordinal nature of the scale, differences between groups were assessed using the Mann–Whitney U test. Statistical analyses were performed using Jamovi software (version 2.3.21.0).

## 3. Results

Regarding pre-intervention outcome variables, no significant differences were found between groups at baseline, suggesting that the groups were comparable before the warm-up intervention. As shown in Table 5, a significant difference was found in the ANOVA for the right shoulder flexion ROM. Bonferroni post hoc analysis indicated a significant increase in the experimental group from T0 to T1 (*p* = 0.008). While no significant difference emerged for the other variables, Borg CR-10 scores, reported in Table 6, were significantly different in the experimental group at T1 (*p* = 0.041).

## 4. Discussion

The present pilot study examined the acute effects of a shoulder warm-up focused on strength and mobility, compared with a usual routine, in young competitive athletes engaged in overhead sports. The main finding was a significant improvement in right shoulder flexion ROM in EG after the strength-based warm-up (*p* = 0.008). No significant effects emerged for left shoulder flexion ROM, handgrip strength, or CKCUES performance. Borg CR-10 scores were also significant after the experimental protocol, suggesting a greater perceived response to the preparatory stimulus. Overall, these findings indicate that the proposed shoulder-focused warm-up may produce a side-specific improvement in right shoulder flexion ROM. Since most participants were right-side dominant, this response may reflect the greater involvement of the dominant limb in repeated overhead sport actions, which may induce side-to-side adaptations in shoulder mobility and muscular control. This is consistent with previous research supporting the hypothesis that overhead athletes may present different ROM profiles and intervention responses between the dominant/throwing and non-dominant shoulders. Accordingly, the greater improvement observed in the right shoulder may represent a more evident acute response of the dominant, sport-adapted limb to the warm-up protocol, rather than a bilateral effect of the intervention [1,35]. At the same time, recent studies have shown that even a single session of stretching or mobility-oriented work can improve range of motion, making the finding observed in the present study plausible within the context of a sport-specific warm-up [36,37]. From an applied perspective, the protocol appears feasible for integration into daily training. In overhead sports, the shoulder complex is exposed to high and repetitive mechanical loads [38]. For this reason, the warm-up should not be considered merely a generic phase at the start of the session. Instead, it may represent an opportunity to prepare the shoulder complex more specifically for the mechanical demands of overhead activity [39,40]. Although the present findings do not allow for conclusions regarding injury prevention, long-term adaptations, or generalized bilateral mobility improvements, they suggest that a targeted warm-up may represent a practical component within daily training routines [41]. This is particularly important for young athletes, who often accumulate many training hours and for whom the practical sustainability of the proposed intervention is a central issue [42]. Recent research on overhead athletes also supports the relevance of simple, specific, and low-cost programs for shoulder function, although preventive effects cannot be inferred from the present acute design [43,44].

The absence of significant differences in CKCUES and handgrip strength may be explained by the nature of these outcomes. CKCUES is more complex than an isolated mobility measure, as it involves upper-limb dynamic stability, trunk control, coordination, and muscular capacity. A single warm-up exposure may therefore have been sufficient to modify ROM, but not a more complex functional task such as CKCUES [45]. This interpretation is consistent with recent research highlighting the usefulness and psychometric properties of CKCUES, while emphasizing that its results should be interpreted in light of its multidimensional nature [46]. In already trained young athletes, this may further reduce the likelihood of observing acute changes.

Similarly, handgrip strength should not be interpreted as a direct marker of shoulder-specific function. In the present study, it was included as a complementary indicator of maximal isometric upper-limb force expression.

However, as a relatively global measure, it may not be sufficiently sensitive to detect subtle acute changes induced by a brief shoulder-specific warm-up. This is consistent with evidence recommending caution when interpreting handgrip strength as an indicator of general strength or functional performance, given the heterogeneous associations with other strength measures across populations and contexts [47].

In trained young athletes, this general measure may not be sensitive enough to detect immediate changes after a single preparatory routine. In line with this caution, recent findings in overhead athletes suggest that differences in scapulohumeral function do not necessarily translate into changes in upper-extremity dynamic stability or handgrip strength [48,49].

Borg CR-10 added a practical element to the interpretation of the present findings, as it was used to assess the athletes’ immediate perceived response to the warm-up. Therefore, the score was interpreted cautiously as an indicator of perceived exertion/internal load, rather than as a direct measure of readiness, satisfaction, or perceived shoulder freedom [50]. The higher values observed after the experimental protocol may suggest that athletes perceived it as a more demanding preparatory stimulus than their habitual warm-up routine. it might be useful underline, this greater perceived response did not appear to reflect fatigue sufficient to impair subsequent performance, as no worsening was observed in the other outcomes and right shoulder flexion ROM improved. This interpretation is consistent with recent evidence supporting RPE as a valid, simple, and practical tool for monitoring internal load, particularly in youth field-based settings where assessment methods should be rapid, sustainable, and easily applicable [51].

## 5. Conclusions

The experimental protocol produced a significant improvement in right shoulder flexion ROM, whereas no significant changes were observed in left shoulder flexion ROM, handgrip strength, or CKCUES performance. Athletes in the experimental group also reported higher Borg CR-10 scores after the warm-up, suggesting a greater perceived response to the preparatory stimulus [52,53]. A sport-specific warm-up may help make the preparatory phase more targeted and relevant to the demands of the subsequent training session, in line with previous research supporting the use of shoulder-focused and specific warm-up strategies in overhead athletes [54,55]. From an applied perspective, this type of protocol could help coaches and athletes use the warm-up as an active part of training, rather than as a generic preliminary phase. However, given the preliminary nature of this pilot study, the protocol should be interpreted as a feasible field-based strategy for shoulder preparation, and not as a standardized practice or as evidence of direct improvements in performance or injury prevention [56]. Future studies with larger samples, longitudinal designs, and more detailed biomechanical, proprioceptive, scapula-specific, and sport-specific performance assessments are needed to better define the practical relevance of this approach and to determine whether repeated use may lead to broader functional or preventive effects.

## 6. Limitations

This study has some limitations that should be considered when interpreting the findings. First, as this was a pilot study, the sample size was relatively small and was determined pragmatically based on the number of eligible athletes available during the study period. This limited the possibility of performing sex-stratified analyses, sport-specific subgroup analyses, or statistical control for modality-related differences. Although participants were recruited from different overhead sport modalities sharing relevant shoulder demands, differences in technical gestures, training history, and loading patterns may have influenced the responses. Biological maturity was also not assessed, although it may influence physical performance and acute responses in youth athletes. Second, the study used feasible field-based outcomes and did not include electromyographic, detailed kinematic, proprioceptive, or scapula-specific assessments. In addition, shoulder mobility was assessed only through shoulder flexion ROM. Therefore, future studies should include larger and more balanced samples, assess all planes of shoulder movement, and incorporate more detailed biomechanical and sport-specific evaluations.

## Figures and Tables

**Table 1:** Anthropometric characteristics.

Variable	EG (n = 12)		SWG (n= 12)	
	Males (n = 3)	Females (n = 9)	Males (n = 5)	Females (n = 7)
Age (years)	16.0 ± 2.65	14.22 ± 0.97	14.80 ± 1.92	15.43 ± 1.27
Height (cm)	164.0 ± 9.54	157.78 ± 5.93	165.0 ± 6.67	154.57 ± 4.16
Body mass (kg)	54.33 ± 13.65	48.0 ± 10.11	59.80 ± 18.06	51.57 ± 5.97

Notes: Data are presented as mean ± standard deviation (mean ± SD); height is reported in centimeters (cm) and body mass in kilograms (kg); EG: experimental group; SWG: habitual warm-up group.

**Table 2:** Overview of assessment tools, measured variables, and study purposes.

Instrument	Variable Measured	Purpose	Administration Time
Gyko	Scapulohumeral joint range of motion	Used to quantify scapulohumeral joint flexion before and after the warm-up intervention	T0–T1
Handgrip	Maximal isometric grip strength	Used as a simple and standardized measure of maximal isometric upper-limb force expression, rather than as a direct marker of shoulder-specific function	T0–T1
CKCUES	Closed kinetic chain functional test assessing upper-limb performance through the number of hand touches performed within a fixed time interval	Used as a field-based functional test to assess upper-limb dynamic stability, trunk contribution, and load tolerance through the upper extremity in a closed kinetic chain	T0–T1
Borg CR-10 (RPE)	Perceived exertion score	Used to evaluate the perceived response and internal load associated with the warm-up	T1

Note: CKCUES: Closed Kinetic Chain Upper Extremity Stability Test; RPE: Rating of Perceived Exertion; T0: before the warm-up; T1: immediately after warm-up.

**Table 3:** Strength- and mobility-based shoulder-focused warm-up protocol.

Exercise	Rounds	Duration/Repetitions	Recovery
Lacrosse ball rolling	3	30″ Each side	No recovery
Shoulder CARs	2	20 Repetitions (10 forward + 10 backward)	30″
Y-Hurdle Raises	2	20 Repetitions	1′
Y Isometric Hold	3	20″ hold	20″

Notes: CARs: Controlled Articular Rotations.

**Table 4:** Traditional warm-up routine.

Exercise	Rounds	Duration/Repetitions	Recovery
Jumping Jacks	3	20 Repetitions	30″
Cat–Cow	2	20 Repetitions	30″
Arm Mobility	2	20 Repetitions	30″
Plank	3	30″ hold	1′

**Table 5:** Shoulder flexion range of motion, handgrip strength, and upper-limb functional performance. (mean ± SD) in the experimental group (EG) and standard warm-up group (SWG) at baseline (T0) and post-warm-up (T1).

Parameters	Group	T0	T1	F	*p*	η^2^p
S. Flexion L. Degrees (°)	EG SWG	168 ± 7.55 161 ± 8.83	172 ± 9.18 162 ± 18.49	2.472	0.130	0.101
S. Flexion R. Degrees (°)	EG SWG	160 ± 8.74 158 ± 9.91	167 ± 9.03 156 ± 14.36	11.63	0.003 *	0.346
Handgrip L (kg)	EG SWG	21.8 ± 4.43 27.8 ± 12.57	23.3 ± 4.55 31.0 ± 10.96	0.0715	0.792	0.003
Handgrip R (kg)	EG SWG	24.2 ± 4.87 30.9 ± 14.85	25.2 ± 4.23 34.3 ± 12.42	2.50	0.128	0.102
CKCUES (repetitions)	EG SWG	13.25 ± 5.55 13.75 ± 6.09	15.92 ± 6.22 16.58 ± 6.64	0.0537	0.819	0.002

Notes: Data are presented as mean ± standard deviation; T0: Baseline evaluation; T1: following warm-up protocol; L: Left; R: Right; S. Flexion: Shoulder flexion; CKCUES: Closed Kinetic Chain Upper Extremity Stability test; F, *p*, and η^2^p values refer to the time × group interaction derived from ANOVA; * statistically significant difference (*p* ≤ 0.05).

**Table 6:** Borg CR-10 perceived exertion (median and IQR) in the experimental group (EG) and standard warm-up group (SWG) at T1.

Parameters	Group	T1	*p*-Value
Borg CR-10 (RPE)	EG	7.25 (7.0–8.0)	0.041 *
	SWG	6.75 (6.0–7.0)	

Notes: Data are presented as the median and interquartile range (IQR). T1: post-warm-up assessment. *p*-value refers to the between-group comparison (Mann–Whitney U test; * statistically significant difference (*p* ≤ 0.05)).

## Data Availability

The data are available on reasonable request from the corresponding author. The data are not publicly available due to privacy and ethical restrictions.

## Abbreviations

The following abbreviations are used in this manuscript:

ROM	Range of Motion
RPE	Rating of Perceived Exertion
CARs	Controlled Articular Rotations
EG	Experimental Group
SWG	habitual warm-up group
CKCUES	Closed Kinetic Chain Upper Extremity Stability Test
SD	Standard Deviation
IQR	Interquartile Range
T0	Baseline Assessment
T1	Post-Warm-Up Assessment
